# Night-time rumination in PTSD: development and validation of a brief measure

**DOI:** 10.1080/20008198.2019.1651476

**Published:** 2019-08-27

**Authors:** Elizabeth Woodward, Juliane Sachschal, Esther T. Beierl, Anke Ehlers

**Affiliations:** a Department of Experimental Psychology, University of Oxford, Oxford, UK; b Oxford Health NHS Foundation Trust, Oxford, UK

**Keywords:** Sleep, posttraumatic stress disorder (PTSD), insomnia, pre-sleep cognitions, rumination, Sueño, Trastorno de estrés postraumático, insomnio, Cogniciones pre-sueño, rumiación, 睡眠, 创伤后应激障碍, 失眠, 睡前认知, 反刍

## Abstract

**Background**: Pre-sleep cognitive activity and arousal have long been implicated in the maintenance of insomnia. However, despite high comorbidity between insomnia and posttraumatic stress disorder (PTSD), pre-sleep thoughts in PTSD and their associations with disturbed sleep, have not yet been investigated.

**Objective**: This study presents the development and preliminary validation of a brief self-report measure of the content of trauma-related pre-sleep thoughts: the Trauma Thoughts before Sleep Inventory (TTSI).

**Methods**: Participants (N = 285) were recruited online into five groups: three groups with clinical symptoms, 1) PTSD; 2) depression without PTSD; 3) insomnia without depression or PTSD; and two healthy control groups 4) nontrauma-exposed controls; 5) trauma-exposed controls. The questionnaire was administered at baseline, and for a subsample (*n =* 157) again one week later to assess test-retest reliability. At baseline, participants also completed questionnaires of sleep quality, PTSD and depression symptoms, and insomnia-related thoughts.

**Results**: The TTSI had good reliability and validity; it discriminated participants with PTSD from those with depression and insomnia, those with depression from insomnia, and correlated with existing measures of pre-sleep thoughts, self-reported pre-sleep arousal and poor sleep.

**Conclusions**: The results support the utility of the TTSI for measuring thoughts that keep people with PTSD awake, although replication in an independent clinical sample is required.

## HIGHLIGHTS

• A new measure was developed and validated to assess trauma-related presleep thoughts.• The new measure discriminated people with PTSD from those with insomnia and depression, and discriminated depression and insomnia.• Trauma-related pre-sleep thoughts in PTSD were associated with measures of sleep and pre-sleep arousal.• Both insomnia-related and trauma-related pre-sleep thought content was associated with poor sleep in PTSD.

## Abstract (es)

**Antecedentes**: La actividad cognitiva y la excitación previa al sueño han estado implicadas durante mucho tiempo en el mantenimiento del insomnio. Sin embargo, a pesar de la alta comorbilidad entre el insomnio y el trastorno de estrés postraumático (TEPT), los pensamientos previos al sueño en el trastorno de estrés postraumático y sus asociaciones con trastornos del sueño, aún no se han investigado.

**Objetivo**: Este estudio presenta el desarrollo y la validación preliminar de una escala breve de autoreporte del contenido de los pensamientos previos al sueño relacionados con el trauma: El Inventario de Pensamientos Traumáticos Previos al Sueño (IPTPS).

**Métodos**: Los participantes (N = 285) fueron reclutados en línea y se dividieron en cinco grupos: tres grupos con síntomas clínicos, 1) TEPT; 2) depresión sin TEPT; 3) insomnio sin depresión o TEPT; y dos grupos de control sanos 4) controles no expuestos a traumas; 5) Controles expuestos al trauma. El cuestionario se administró al inicio del estudio y para una submuestra (n = 157) nuevamente una semana después, para evaluar la confiabilidad test-retest. Al inicio, los participantes también completaron cuestionarios de calidad del sueño, trastorno de estrés postraumático y depresión, y pensamientos relacionados con el insomnio.

**Resultados**: El IPTPS tuvo buena confiabilidad y validez; discriminó a los participantes con TEPT de aquellos con depresión e insomnio, aquellos con depresión de los con insomnio y se correlacionó con las medidas existentes de los pensamientos previos al sueño y auto-reporte de activación previa al sueño y mala calidad de sueño.

**Conclusiones**: Los resultados apoyan la utilidad del IPTPS para medir pensamientos que mantienen despiertas a las personas con TEPT, aunque se requiere la replicación en una muestra clínica independiente.

## Abstract (zh)

**背景：**长期以来，睡眠前认知活动和唤醒一直与失眠的维持有关。然而，尽管失眠与创伤后应激障碍（PTSD）之间存在高度共病，创伤后应激障碍中的睡前想法及其与睡眠障碍的关系尚未得到研究。

**目的：**本研究呈现了一个测量有关创伤的睡前想法内容的简短自评工具：《入睡前创伤想法量表》（TTSI）的发展和初步验证。

**方法：**将在线招募的285名参与者分为五组：三组有临床症状：1）创伤后应激障碍组; 2）无创伤后应激障碍的抑郁症组; 3）无抑郁或创伤后应激障碍的失眠组;和两个健康对照组4）非创伤暴露对照组; 5）创伤暴露对照组。问卷在基线时施测，并在一周后再测了157名参与者以评估重测信度。在基线时，参与者还完成了睡眠质量、创伤后应激障碍和抑郁症状以及与失眠有关的想法的问卷调查。

**结果：**TTSI具有良好的信度和效度;它能区分出PTSD患者与患有抑郁症和失眠的患者，也能区分抑郁症患者和失眠患者，并把睡前想法的现有测量与自我报告的睡眠前唤醒和睡眠质量不良建立了关联。

**结论：**尽管仍需要在独立的临床样本中重复，此结果支持了TTSI可用于测量使 PTSD 患者无法入睡的想法。

## 1. Introduction

There is good evidence for the role of pre-sleep cognitions in maintaining insomnia (see Harvey, 2002). For example, ‘an over-active mind’ is one of the most common reasons given by insomniacs to account for poor sleep (Espie, Lindsay, Brooks, Hood, & Turvey, 1989), and excessive pre-sleep cognitive activity has been associated with longer sleep onset latency and shorter sleep duration (Gross & Borkovec, 1982). Further, there is evidence that while trying to sleep, insomniacs are more pre-occupied with thoughts about getting to sleep and the consequences of poor sleep (Wicklow & Espie, 2000), as opposed to the ‘nothing in particular’ commonly reported by good sleepers (Harvey, 2010). Pre-sleep thoughts of this kind as well as those of ‘rehearsal and planning’ (e.g. ‘what happened today and what I’ve got on tomorrow’) (Espie et al., 2014) predict longer sleep onset latencies (Harvey & Espie, 2004), and are more frequent in insomniacs versus controls (Harvey & Espie, 2004). Accordingly, most models of insomnia propose that cognitive processes such as cognitive arousal, worry and maladaptive beliefs about sleep and its consequences play a role in maintaining insomnia (Espie et al., 1989; Lundh & Broman, 2000).

Insomnia is highly prevalent in PTSD (Ohayon & Shapiro, 2000) and is implicated in PTSD development and maintenance (see Babson & Feldner, 2010). It has been suggested that factors known to perpetuate general insomnia may also contribute to sleep impairment in PTSD (Zayfert & DeViva, 2004). Pre-sleep thoughts may be one candidate factor, but have not yet been directly investigated in PTSD. However, there are some findings that point to a role of fear-related cognitions. For example, studies have found that fear of sleep (Kanady et al., 2018; Pruiksma et al., 2014; Short, Allan, Stentz, Portero, & Schmidt, 2018) linked to nightmares (Davis et al., 2011; Krakow, Tandberg, Scriggins, & Barey, 1995), and fear of loss of vigilance (Pietrzak, Morgan, & Southwick, 2010) are associated with worse sleep and more severe PTSD symptoms in trauma survivors. These findings are consistent with relationships proposed in the maintenance of PTSD symptoms by Ehlers and Clark’s (2000) cognitive model of PTSD. This model proposes that trauma-related appraisals related to impending danger (such as ‘I will be attacked again’) or negative interpretations of symptoms (such as ‘If I go to sleep I will not notice intruders’ (p. 330) or ‘Not sleeping will damage my body’) motivate the use of dysfunctional coping strategies such as staying up late because of fear of nightmares, or ruminative thoughts about the trauma, or repetitive thinking about one’s emotional state (similar to brooding about feeling sad in depression, Nolen-Hoeksema, 1991), which may then interfere with sleep onset and quality (Ehlers & Clark, 2000). Consistent with this, in non-clinical populations, maladaptive repetitive thoughts have been linked to more severe sleep disturbances (Cox, Ebesutani, & Olatunji, 2016; Nota & Coles, 2015), and shown to mediate the relationship between depressed mood and sleep quality (Slavish & Graham-Engeland, 2015). Studies in clinical populations of individuals with Generalised Anxiety Disorder have also shown that the frequency of evening worry predicts subsequent sleep quality, and vice versa (Thielsch et al., 2015), and rumination following a stressor has been found to predict increased self-reported and objective sleep onset latency (Zoccola, Dickerson, & Lam, 2009). Finally, in trauma survivors, rumination has been linked to worse sleep problems and PTSD symptoms, and is suggested to contribute to impaired sleep (Borders, Rothman, & McAndrew, 2015). It is thus possible that trauma-related pre-sleep thoughts (such as appraisals of being vulnerable while asleep, or repetitive thoughts about how the trauma could have been prevented or about negative feelings) play a role in maintaining sleep problems in PTSD. These trauma-related pre-sleep thoughts could occur alongside pre-sleep thoughts already evidenced in insomnia populations (such as worries about getting enough sleep) (Harvey, 2002; Harvey & Espie, 2004), and both could contribute to poor sleep in PTSD.

The Glasgow Content of Thought Inventory (GCTI; Harvey & Espie, 2004) was developed to assess pre-sleep thought patterns in insomnia populations. Items were generated from pre-sleep thoughts reported by people with insomnia, and so might not assess the full range of pre-sleep thoughts that could interfere with sleep in PTSD. As no measure currently exists to assess pre-sleep thought patterns that are specific to PTSD, it would therefore be of interest to develop such a measure, and to investigate its association with disturbed sleep in PTSD.

### 1.1. Aims

This study describes the development and initial validation of a new measure designed to assess trauma-related pre-sleep thought patterns that may interfere with sleep in people with PTSD; the Trauma Thoughts before Sleep Inventory (TTSI).

The specific aims of the present study were to 1) develop a measure of trauma-related pre-sleep thoughts, 2) to assess the psychometric properties of the new measure, and 3) to investigate whether trauma-related pre-sleep thoughts discriminate people with PTSD from those with insomnia or depression, and from traumatised and non-traumatised controls. Due to the high comorbidity between PTSD and depression (Kessler, Chiu, Demler, & Walters, 2005), and the high prevalence of insomnia in depression (Riemann, Berger, & Voderholzer, 2001) individuals with depression were included as a comparison group to determine whether the trauma-related pre-sleep thought patterns were specific to PTSD.

## 2. Methods

An online study evaluated the reliability of a new questionnaire of trauma-related pre-sleep thoughts and examined its associations with established measures of sleep disturbance, arousal, and PTSD symptoms and its specificity to PTSD compared to depression and insomnia.

### 2.1. Participants

A sample of 285 participants aged 18–65 (*M* = 30.32 years, *SD* = 11.37) took part in the study between 2013 and 2016 (see Table 1 for demographics). This included 159 women (55.8%). Inclusion criteria for all groups were that they were aged 18–65, could read and write in English, reported no present or past diagnosis of psychosis or bipolar disorder, reported no present substance or alcohol dependence, and were not currently receiving psychological therapy (as this may have affected the test-retest reliability analyses).

**Table 1.** Demographic and trauma information for each group.

	Control	Trauma	PTSD	Depression	Insomnia
	(*n* = 80)	(*n* = 77)	(*n* = 57)	(*n* = 29)	(*n* = 42)
	*M (SD)*	*M (SD)*	*M (SD)*	*M (SD)*	*M (SD)*
**Age (years)**	27.23 (9.04)	32.56 (12.31)	33.90 (11.69)	30.75 (15.98)	25.84 (6.81)
	***n* (%)**	***n* (%)**	***n* (%)**	***n* (%)**	***n* (%)**
**GENDER**					
*Women*	49 (61.3)	43 (55.8)	30 (52.6)	16 (55.2)	23 (54.8)
*Men*	31 (38.8)	34 (44.2)	27 (47.4)	13 (44.8)	19 (45.2)
**ETHNIC BACKGROUND**					
*Caucasian*	32 (88.8)	47 (84.0)	37 (75.0)	13 (76.5)	21 (91.4)
*Ethnic minority*	4 (11.2)	9 (16.0)	12 (25.0)	4 (23.5)	2 (8.6)
*Missing data*	44	21	8	12	19
**TRAUMA CHARACTERISTICS (*n*)**					
**Traumatised**	0	77 (100)	57 (100)	11 (37.9)	7 (16.7)
*Non-interpersonal*	—	57 (72.0)	32 (65.1)	7 (66.7)	5 (71.5)
*Interpersonal*	—	20 (27.4)	22 (40.9)	4 (33.4)	2 (28.6)
*Missing*	—	4	3	0	0
**RETEST QUESTIONNAIRE**					
Completed	64 (81.0)	57 (73.1)	18 (31.6)	10 (34.5)	8 (19.0)

Trauma characteristics are for those who had experienced a trauma (n = 152). Interpersonal trauma includes non-sexual assault, sexual assault, torture and child abuse. Non-interpersonal trauma includes road accident, accident/natural disaster, witnessing others die, sudden traumatic loss and ‘other’.

Participants were recruited by adverts and sent a screening questionnaire including measures of trauma exposure, PTSD symptoms, depression, insomnia and general questions about their mental health. On the basis of the self-reported symptoms in standardised questionnaires, participants were divided into one of five groups. Three groups reported clinically significant symptoms, using empirically established cut-offs:

1. PTSD: These participants reported at least one traumatic event and a PTSD symptom severity of 18 or above (Ehring, Kleim, Clark, Foa, & Ehlers, 2007) on the Posttraumatic Diagnostic Scale (Foa, Cashman, Jaycox, & Perry, 1997) (*n* = 57).
2. Depression: These participants reported depression symptom severity of 10 or above on the Patient Health Questionnaire (PHQ-9; Kroenke, Spitzer, & Williams, 2001), but no significant PTSD symptoms (*n* = 29).
3. Insomnia: These participants reported insomnia severity of 10 or above (Morin, Belleville, Bélanger, & Ivers, 2011) on the Insomnia Severity Index (ISI; Bastien, Vallieres, & Morin, 2001), but no significant depression or PTSD symptoms (*n* = 42).

Two control groups:

1. Controls: These participants reported no trauma and no depression, insomnia, PTSD, and did not report any other mental health diagnoses (*n* = 80).
2. Trauma controls: These participants reported at least one trauma, but did not report depression, insomnia, PTSD, and did not report any other mental health diagnoses (*n* = 77).

Of 420 advert respondents, 354 people (84.29%) were suitable for one of the groups, and 285 (80.51%) completed the first online TTSI questionnaire. One week later, 157 of these participants (55.10%) completed the questionnaire again to assess test-retest reliability (Table 1).

### 2.2. Initial development of the trauma thoughts before sleep inventory

The item-pool for the *Trauma Thoughts before Sleep Inventory (TTSI)* was developed from the literature, and through discussions with PTSD patients and clinical psychologists experienced in the treatment of PTSD. Respondents rated how often each thought had kept them awake (when they were trying to fall asleep, or when they had woken in the night) over the previous week, on a scale from 0 (*Not at all)* to 3 (*5 or more times a week/nearly every day*). The initial pool of items was refined through informal interviews and feedback after completion of the scale by a small sample of PTSD patients, healthy trauma survivors, and healthy controls, to check for understanding and repetition. The final item pool comprised 9 items representing empirically and theoretically supported possible trauma-related pre-sleep thoughts (e.g. ‘being vulnerable if I sleep’). The total score is the sum of the items (range: 0–27); higher scores indicated greater frequency of pre-sleep thoughts (see Figure 3 for scale).

### 2.3. Validation measures

To examine the validity of the TTSI, participants also completed the following measures:

#### 2.3.1. Pre-sleep cognitive activity and arousal

*Glasgow Content of Thoughts Inventory (GCTI;* Harvey & Espie, 2004); The GCTI is a 25-item measure of pre-sleep thoughts that have been shown to be linked to insomnia. Respondents rate how often each thought kept them awake over the previous 7 days on a 4-point scale from *never (= 1)* to *always (= 4)* (range: 25–100). The scale has good psychometric properties (Cronbach’s α = .87; Harvey & Espie, 2004). To test whether TTSI items constitute a separate factor from general insomnia-related thoughts, 13 non-trauma related items were adapted from the GCTI (see Table 3) and rated on the same rating scale as the TTSI.

**Table 2.** Descriptive statistics for each group, and test-statistics for group comparisons. Group differences are indicated in superscript (e.g. ^A, B, C,^), groups sharing the same letter do not differ.

	Control	Trauma	PTSD	Depression	Insomnia	
	(*n* = 80)	(*n* = 77)	(*n* = 57)	(*n* = 29)	(*n* = 42)	Group DIFF
	*M (SD)*	*M (SD)*	*M (SD)*	*M (SD)*	*M (SD)*	*F*/H
**MOOD AND ANXIETY**						
BDI	3.82 (4.63)^B^	5.29 (4.77)^B^	23.32 (8.76)^A^	21.48 (7.94)^A^	6.78 (4.85)^B^	H = 148.08***
GAD-7	2.78 (3.22)^C^	2.05 (1.81)^C^	12.50 (5.63)^A^	10.48 (4.81)^A^	5.59 (3.96)^B^	H = 59.00***
PDS (n = 152)		6.75 (5.66)^C^	35.24 (10.88)^A^	16.09 (7.66)^B^	10.71 (6.52)^B,C^	H = 131.24***
**SLEEP**						
PSQI global score	3.82 (1.79)^C^	4.78 (2.82)^C^	10.77 (3.27)^A^	8.14 (3.23)^B^	7.78 (2.74)^B^	F = 66.75***
Component 2 (SOL)	16.07 (11.17)^B^	20.89 (20.41)^B^	60.96 (64.42)^A^	38.90 (28.32)^A^	40.76 (28.52)^A^	F = 22.16***
PSQI (Addendum for PTSD)	1.84 (2.08)^C^	2.71 (2.13)^C^	7.77 (3.92)^A^	4.72 (3.26)^B^	3.83 (3.51)^B^	H = 94.39***
**PRE-SLEEP AROUSAL**						
PSAS-cog	13.45 (4.99)^C^	14.35 (5.11)^C^	25.43 (8.02)^A^	20.55 (6.70)^A,B^	19.30 (7.10)^B^	H = 92.73***
PSAS-som	9.29 (1.89)^C^	9.85 (2.62)^C^	17.19 (7.51)^A^	12.76 (4.99)^A,B^	10.70 (3.25)^B,C^	H = 67.38***
**PRE-SLEEP THOUGHTS**						
Trauma-related (TTSI)	0.98 (1.80)^C^	1.30 (2.00)^C^	9.70 (6.85)^A^	4.28 (3.83)^B^	2.53 (2.78)^B,C^	H = 112.47***
GCTI (total score)	37.02 (9.51)^B^	38.32 (9.18)^B^	58.55 (16.74)^A^	51.48 (15.06)^A^	47.95 (12.52)^A^	H = 84.22***

BDI = Beck Depression Inventory; GAD-7 = Generalised Anxiety Disorder Scale; PDS = Posttraumatic Diagnostic Scale; PSQI = Pittsburgh Sleep Quality Index; SOL = Sleep onset latency; PSAS-cog = Pre-sleep arousal scale, cognitive subscale; PSAS-som = Pre-sleep arousal scale somatic sub-scale; TTSI = Trauma Thoughts before Sleep Inventory; GCTI = Glasgow Content of Thought Inventory. PDS is for trauma survivors only (n = 152). Significance level of group difference indicated by: * p < .05; ** *p* < .01, *** *p* < .001.

**Table 3.** Two factor solution with Oblimin rotation.

	Extracted factors	
**Original Item Pool**	**1**	**2**
That I am still not asleep	0.99	
How long I have been awake	0.84	
That I won’t be able to cope with so little sleep	0.75	
How getting too little sleep affects me	0.70	
How tired‎/sleepy I feel	0.54	
Checking the time	0.58	
Everyday worries (e.g. about work or home life)	0.68	
Tomorrow (plans, things I have to do)	0.72	
How I can’t stop my mind from racing	0.73	
How restless I feel	0.54	
Worries about the more distant future	0.60	
Things that happened during the day	0.58	
Noises I hear	—	**—**
**Unwanted memories of the event**		**0.61**
**Dwelling on the event (e.g. asking yourself ‘why me,’ ‘what if’ questions)**		**0.63**
**Dwelling on how my life has been changed by the event**		**0.51**
**Being vulnerable if I sleep**		**0.86**
**How afraid I feel**		**0.77**
**Something bad happening to my body if I sleep (e.g. heart attack, stop breathing, will not wake up, will die)**		**0.65**
**Fear of nightmares**		**0.44**
**How lonely I feel**		**0.61**
**How angry I feel**		**0.49**

Factor 1 = GCTI-adapted; Factor 2 = Trauma Thoughts before Sleep Inventory (TTSI); **TTSI items are indicated in bold.**

*Pre-Sleep Arousal Scale (PSAS)* (Nicassio, Mendlowitz, & Fussell, 1985). The PSAS is a 16-item scale with two sub-scales (8 items each) measuring the intensity of somatic (PSAS-som) and cognitive (PSAS-cog) arousal, while trying to fall asleep. Items are rated on a scale of 0 (*not at all*) to 5 (*extremely*) (range: 0–40). Both sub-scales have good psychometric properties (PSAS-cog; α = .76; PSAS-som; α = .81) in individuals with insomnia (Nicassio et al., 1985).

#### 2.3.2. Sleep symptoms

*Pittsburgh Sleep Quality Index (PSQI;* Buysse, Reynolds, Monk, Berman, & Kupfer, 1989) The PSQI is a widely used 19-item scale, which assesses seven components of sleep quality and disturbances over the previous month. Items and sub-scales are scored from 0 (*no difficulty*) to 3 (*severe difficulty*) and summed to produce a PSQI global score (range: 0–21; higher scores indicate more severe global sleep disturbances). A global score of greater than 5 can be used to indicate clinically significant sleep disturbances (Buysse et al., 1989). To assess time taken to fall asleep, PSQI Component 2 was used (Sleep Onset Latency (SOL); range: 0–3). The *PSQI Addendum for PTSD (PSQIA)* (Germain, Hall, Krakow, Shear, & Buysse, 2005) was also included as a measure of the severity of PTSD related sleep disturbances over the past month (range: 0–21).

#### 2.3.3. Trauma exposure and PTSD symptoms

*Life Events Checklist (LEC)*. The LEC (Gray, Litz, Hsu, & Lombardo, 2016) is a self-report questionnaire containing a list of stressful and traumatic (according to DSM-5 criteria) life events. Respondents indicate whether they have experienced each event in their lifetime. Participants also answered questions about the worst event they had experienced, to determine if it met DSM-5 criteria for a traumatic event (Criterion A; American Psychiatric Association, 2013).

*Posttraumatic Diagnostic Scale* (PDS) (Foa et al., 1997). The PDS is a validated self-report measure assessing the severity of DSM specified PTSD symptoms over the previous week, rated on a scale from 0 (*not at all*) to 3 (*5 or more times a week/nearly every day)*. A cut-off of 18 has been found to show the greatest diagnostic accuracy in predicting a PTSD diagnosis according to the Structured Clinical Interview for DSM-IV (Ehring et al., 2007). In the present study, the Clinician Administered PTSD Scale for DSM-5 (CAPS; Weathers et al., 2013) was administered by one of the authors (EW or JS) at Week 1 for a sub-set of trauma controls (*n* = 36) and participants with PTSD (*n* = 40). Cohen’s kappa showed ‘very good’ agreement (94.29%) between the CAPS and the PDS cut off, κ = .87, *p* < .001.

#### 2.3.4. Mood and anxiety symptoms

*Beck Depression Inventory* (BDI; Beck, Ward, Mendelson, Mock, & Erbaugh, 1961); a 21-item questionnaire assessing depressive symptoms over the previous two weeks (range: 0–63; higher scores indicate more severe depression).

*Generalised Anxiety Disorder Scale* (GAD-7; Spitzer & Kroenke, 2006); a 7-item questionnaire assessing the frequency of general anxiety symptoms over the previous week (range: 0–21; higher scores indicate more severe anxiety).

### 2.4. Procedure

#### 2.4.1. Screening and allocation

Participants were recruited via different online adverts in the community in Oxford and at the University of Oxford. Adverts invited people with either (1) difficulties falling or staying asleep, (2) low mood, (3) experience of a trauma, or (4) healthy controls to contact the research team. Advert respondents completed online screening questionnaires, which consisted of the LEC (Gray et al., 2016) to identify trauma exposure, the PDS (Foa et al., 1997) to assess PTSD symptoms (for those who reported a traumatic event), and questions about their current and past mental health. Participants responding to adverts for low mood or sleeping problems (in the past two weeks) also completed the PHQ-9 (Kroenke et al., 2001), and the ISI (Bastien et al., 2001) as screening measures for depression and insomnia, respectively. Suitable participants were allocated to a group and emailed the online baseline questionnaire pack.

#### 2.4.2. Baseline questionnaire

Participants completed the questionnaires in a fixed order. First, participants completed the LEC again and identified the worst event they had experienced from those reported, then answered questions to determine if the event met criteria for a traumatic event (Criterion A) according to DSM 5 criteria. Participants were then asked to answer the PTSD symptom measure (PDS) with the ‘traumatic event’ they had identified in mind. They then completed the PSQI (and addendum), GCTI, PSAS and the TTSI. Finally they completed the BDI and GAD-7 to assess mood and anxiety. Non-trauma-exposed participants were asked to answer the PDS, TTSI and PSQIA with the ‘worst event’ they had identified in mind, and the wording was amended to ‘the event’ rather than ‘the trauma/traumatic event/experience’.

#### 2.4.3. One-week questionnaire

After completing the baseline questionnaire pack (Week 1), the TTSI was emailed again one week later (Week 2), to examine test-retest reliability.

### 2.5. Analysis

Analyses were conducted using SPSS version 22. Data was checked for skewness and kurtosis, and homogeneity of variance, and parametric and non-parametric tests were used where appropriate.

#### 2.5.1. Reliability

Internal consistency was evaluated with Cronbach’s alpha (α > 0.8 indicates good internal consistency; Nunnally & Bernstein, 1994), calculated for the entire sample and the PTSD group. For participants who completed the TTSI a second time, the mean sum score at Week 1 and Week 2 was correlated (intraclass correlation) to examine test-retest reliability.

#### 2.5.2. Validity

Factorial validity of the new measure was evaluated with Exploratory Factor Analysis (Principal Axis Factoring), to explore whether pre-sleep thoughts related to insomnia and trauma were represented by two separate factors in the PTSD sample only. The Kaiser-Meyer-Olkin criterion (0.84) and the Bartlett test (χ^2^(231) = 822.11, p < .001) indicated that data were highly suitable for factor analysis. Factors were rotated using oblique rotation (Oblimin method). Factor extraction was supported by parallel analysis (Horn, 1965), which suggests extracting factors whose eigenvalues are bigger than 95% of random eigenvalues, taking into account the sample size and number of items. Item retention was based on factor loadings larger than 0.4 and differences between primary and secondary loadings not smaller than 0.2, to determine the most interpretable factor solution (Matsunaga, 2015).

To examine convergent validity, the new measure was correlated with existing self-report measures of pre-sleep cognitive activity in insomnia (GCTI); sleep quality (PSQI); sleep onset latency (SOL; PSQI Component 2), and pre-sleep arousal (PSAS). Pre-sleep cognitive activity (GCTI) in patients with insomnia (Harvey & Espie, 2004) has been previously correlated with increased pre-sleep mental arousal (PSAS-cog) and increased self-reported sleep onset latency (Harvey & Espie, 2004), therefore these arousal measures were also used to evaluate the construct validity of the TTSI. In trauma survivors only (*n* = 152), the TTSI was correlated with PTSD symptom severity excluding the sleep item (PDSwos).

To investigate criterion validity, the ability of the TTSI to discriminate between experimental groups was examined using multinomial regressions. For comparison, the ability of the GCTI to discriminate between groups was also examined.

#### 2.5.3. Logistic regression

The contribution of trauma- and insomnia-related pre-sleep thoughts to poor sleep in trauma survivors was examined using logistic regression. Sum scores from the TTSI and GCTI were entered as continuous predictors of sleeper status according to the PSQI, where a global score greater than 5 was taken to indicate poor sleep.

## 3. Results

### 3.1. Symptom severity

The severity of sleep, mood and PTSD symptoms for the five groups is shown in Table 2. The severity of sleep disturbances (PSQI global) was compared across groups using a one-way ANOVA. There was a significant main effect of group. After correcting for multiple comparisons, people with PTSD scored significantly higher on the PSQI compared to all other groups (*p*’s < .001); mean difference compared to depression = 2.55 (95% Confidence Interval (CI) = 0.86 to 4.23), *p* < .001; insomnia = 2.92 (95% CI [1.42, 4.42]), *p* < .001; non-exposed controls = 6.82 (95% CI [5.54, 8.11]), *p* < .001; and trauma-exposed controls = 5.97 (95% CI [4.68, 7.25]), *p* < .001. Insomnia and depression did not differ from each other (*p* > .05). The depression group had higher PSQI scores than controls, mean difference = 4.27 (95% CI [2.67, 5.88]), *p* < .001; and trauma-exposed controls = 3.42 (95% CI [1.82, 5.02]), *p* < .001. People with insomnia also had higher PSQI scores than non-exposed controls, mean difference = 3.90 (95% CI [2.49, 5.31]), *p* < .001; and trauma-exposed controls = 3.04 (95% CI [1.63, 4.46]). The PSQIA showed the same pattern of results (Table 2).

### 3.2. Internal consistency

Internal consistency was high for the TTSI (α = 0.90; N = 285; PTSD sample α = 0.89; *n* = 57), and could not be improved with the deletion of items. The corrected item-total correlation was high (*r = *.68; range: *r* = .59-.78; PTSD sample *r* = 0.64; range: *r* = 0.51–0.74).

### 3.3. Test-retest reliability

The scale had good test-retest reliability, scores at Week 1 significantly correlated with scores at Week 2, ICC = .75, *p* < .001, (95% CI [0.67, 0.81]); PTSD ICC = 0.58, *p* < .01, indicating good reliability. A smaller percentage of the three clinical groups completed the follow-up questionnaire. However completers vs. non-completers did not differ on baseline PDS, BDI, PSQI, GCTI, or TTSI for any group (*p*’s > 0.05).

### 3.4. Factorial Validity

Principal axis factor analysis was conducted on TTSI items together with the 13 insomnia-related items adapted from the GCTI (Table 3), using the PTSD sample *(n =* 57). Parallel analysis suggested extracting two factors, which explained 54.45% of the overall variance. Factor 1 (Eigenvalue = 9.78, Percentage of explained variance 44.45%) could be interpreted as insomnia-related thoughts (GCTI-adapted items) and Factor 2 (Eigenvalue = 2.20, Percentage of explained variance 10.00%) as trauma-related thoughts (TTSI items) (see Table 3). The two factors correlated highly, *r* = .54, *p* < .001. Item 3 (‘noises I hear’) did not substantially load on any of the two factors. No substantial cross-loadings were observed.

### 3.5. Convergent validity

Spearman’s rank correlations showed that the TTSI significantly correlated with sleep quality (PSQI global), *r = *.61, *p* < .001; subjective sleep onset latency (PSQI subscale), *r = *.40, *p* < .001; insomnia-related pre-sleep thoughts (GCTI), *r* = .66, *p < *.001, pre-sleep cognitive arousal (PSAS-cog), *r = *.65, *p* < .001, and somatic arousal (PSAS-som), *r = *.61, *p* < .001. In trauma survivors (*n* = 152), the TTSI was also strongly associated with PTSD symptom severity (PDSwos), *r = *.83, *p* < .001 and PTSD-related sleep disturbances (PSQIA), *r* = .69, *p* < .001.

### 3.6. Criterion validity

#### 3.6.1. Group comparison: TTSI

**Figure 1.**
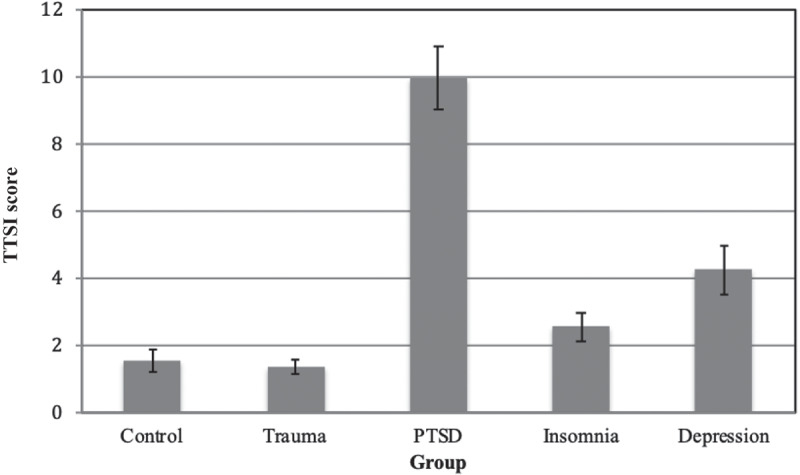
Mean Trauma Thoughts before Sleep Inventory (TTSI) scores and standard error bars for each group (N = 285).

Multinomial regressions showed that greater TTSI scores were associated with a higher likelihood of being in the PTSD group compared to all other groups (non-exposed controls, *b (SE)* = 0.48 (0.07), *exp. b* = 0.62, *p* < .001; trauma-exposed controls, *b (SE)* = 0.55 (0.08), *exp. b* = 0.58, *p* < .001; insomnia, *b (SE)* = 0.33 (0.07), *exp. b* = 0.72, *p* < .001; depression, *b (SE)* = 0.18 (0.06), *exp. b* = 0.83, *p* < .001). Reporting more trauma-related thoughts was also associated with an increased likelihood of being in the depression or insomnia group compared to the non-exposed control group (depression, *b (SE)* = 0.30 (0.07), *exp. b* = 1.35, *p* < .001; insomnia, *b (SE)* = 0.15 (0.07), *exp. b* = 1.16, *p* < .05) and compared to the trauma-exposed control group (depression, *b (SE)* = 0.37 (0.08), *exp. b* = 1.44, *p* < .001; insomnia, *b (SE)* = 0.22 (0.08), *exp. b* = 1.25, *p* < .001). Scoring higher on the TTSI was also related to a higher likelihood of being in the depression group compared to the insomnia group, *b (SE)* = 0.15 (0.07), *exp. b* = 1.16, *p* < .05. There was no difference between the trauma-exposed and non-exposed control groups, *b (SE)* = 0.07 (0.08), *exp. b* = 1.07, *p* > .05 (see Figure 1 and Table 2). All significant comparisons showed medium to large effect sizes (Cohen, 1992).

**Figure 2.**
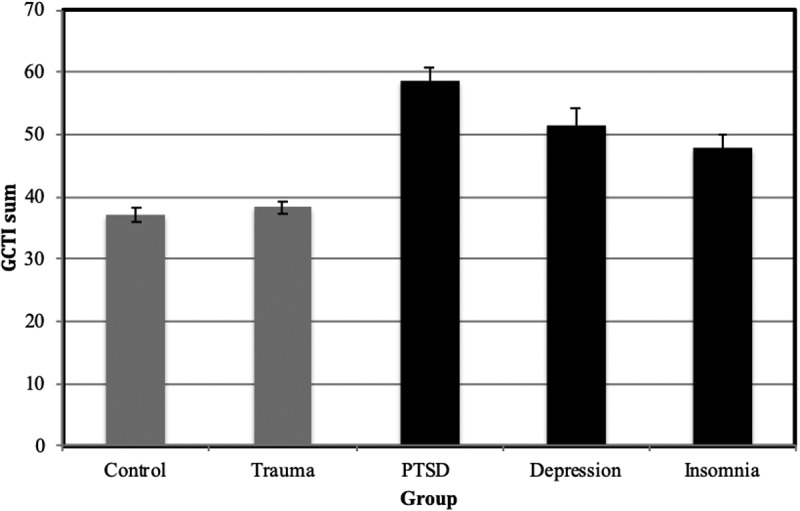
Mean total Glasgow Content of Thoughts Inventory (GCTI) score with standard error bars for each group (N = 285). Clinical groups are indicated in black.

#### 3.6.2. Group comparison: glasgow content of thought inventory

For comparison, groups were compared on the GCTI (see Figure 2). Greater scores on the GCTI were associated with a higher likelihood of belonging to the PTSD group compared to all the other groups (non-exposed controls, *b (SE)* = 0.13 (0.02), *exp. b* = 0.85, *p* < .001; trauma-exposed controls, *b (SE)* = 0.12 (0.02), *exp. b* = 0.85, *p* < .001; insomnia, *b (SE)* = 0.05 (0.01), *exp. b* = 0.94, *p* < .01; depression, *b (SE)* = 0.03 (0.02), *exp. b* = 0.94, *p* < .05). Greater GCTI scores were also associated with an increased likelihood of being in one of the other two clinical groups compared to the non-exposed control group (insomnia, *b (SE)* = 0.08 (0.02), *exp. b* = 1.11, *p* < .001; depression, *b (SE)* = 0.10 (0.02), *exp. b* = 1.11, *p* < .001) and to the trauma-exposed control group (insomnia, *b (SE)* = 0.07 (0.02), *exp. b* = 1.11, *p* < .001; depression, *b (SE)* = 0.08 (0.02), *exp. b* = 1.11, *p* < .001). Magnitude of likelihoods was not significantly different between the depression and insomnia groups, *b (SE)* = 0.01 (0.02), *exp. b* = 1.01, *p* > .05, and between the trauma-exposed and non-exposed control group, *b (SE)* = 0.01 (0.02), *exp. b* = 1.00, *p* > .05. Effect sizes of the significant group differences were small to medium.

**Figure 3.**
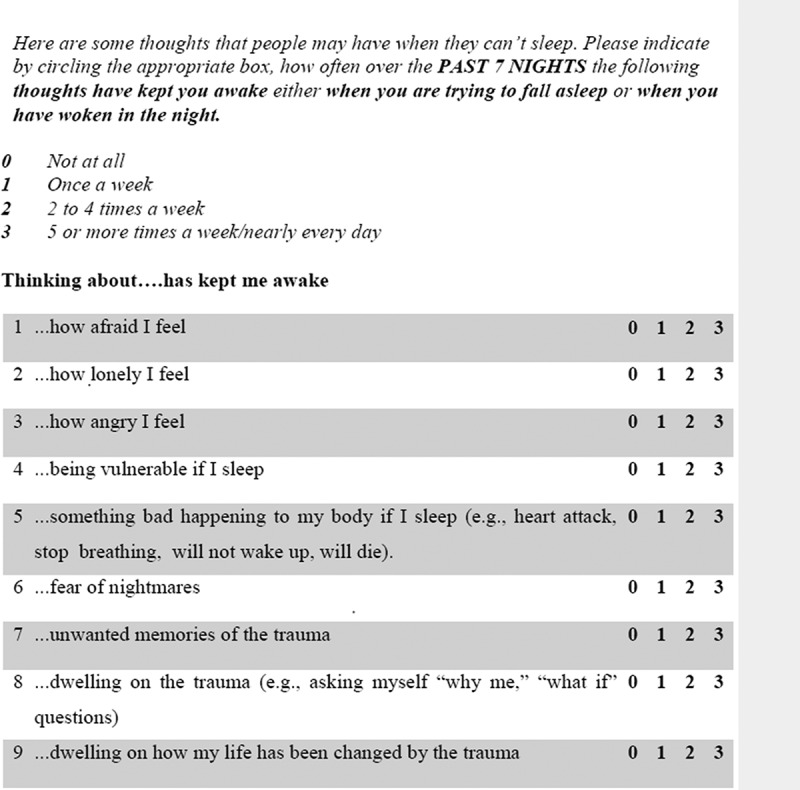
Trauma Thoughts before Sleep Inventory (TTSI).

### 3.7. Predicting poor sleeper status in trauma survivors

A logistic regression was performed to identify the effects of insomnia (GCTI) and trauma-related thoughts (TTSI) on poor sleep in trauma survivors (*n* = 77). The model was statistically significant χ^2^(2) = 91.05, *p* < 0.001, and correctly classified 82.1% of cases, Nagelkerke *R^2^* = .61. Both greater trauma-related and insomnia-related thoughts were associated with increasing likelihood of poor sleep (Table 4).

**Table 4.** Logistics regression results; the impact of trauma-related (TTSI) and insomnia-related (GCTI) thoughts on poor sleep (PSQI).

Measure	Exp. B	SE	Sig.	95% CI
TTSI	1.30	0.09	0.005	1.08–1.56
GCTI	1.14	0.03	0.000	1.08–1.21

## 4. Discussion

This study presented the development and initial validation of a measure designed to assess trauma-related thoughts that may interfere with sleep (e.g. ‘*Being vulnerable if I sleep’*, or ‘*dwelling on how my life has been changed by the event’)*. It was also explored whether these thoughts discriminated people with PTSD from those with insomnia and depression, to determine the TSSI’s utility as a tool to identify trauma- and PTSD-related pre-sleep thought content, which might contribute to poor sleep in PTSD.

The TSSI had good psychometric properties, including good test-retest reliability and internal consistency, and good validity, correlating highly with the GCTI, an existing validated measure of pre-sleep cognitive activity in insomnia (Harvey & Espie, 2004).

The TTSI showed good convergent validity. Participants with a greater frequency of trauma-related thoughts before sleep reported higher pre-sleep arousal on the PSAS (both somatic and cognitive) and more severe sleep difficulties (longer sleep onset, and worse sleep quality) on the PSQI. This is consistent with previous findings that pre-sleep cognitive activity is associated with insomnia (Harvey, 2002, 2003, 2010; Harvey & Espie, 2004), and interferes with sleep by prolonging sleep onset latency (Gross & Borkovec, 1982). The results of this study could similarly suggest that in PTSD there are trauma-related thought patterns associated with elevated pre-sleep arousal and worse sleep (perhaps by prolonging sleep onset). However, the present results do not indicate a causal relationship.

The TTSI also showed good criterion validity. The PTSD group reported more frequent trauma-related thoughts before sleep than any other group. The TTSI discriminated people with PTSD from other clinical groups associated with poor sleep (depression and insomnia). It also discriminated depression from insomnia, whereas the GCTI did not. This could be explained by a larger number of trauma survivors in the depression versus insomnia group, who may be more likely to ruminate on the trauma before sleep, but do not differ on PTSD-related sleep complaints (PSQIA) and PTSD symptoms compared to the insomnia group. It further suggests that, in addition to PTSD, the TTSI could also be relevant to understanding the symptom experience in other trauma and stressor-related disorders, and therefore may have wider clinical applications; for example with individuals with depression following a traumatic stressor.

More frequent insomnia-related thoughts before sleep (GCTI) were also more likely to be associated with PTSD than the other groups, although to a lesser extent than trauma-related thoughts. People with depression and insomnia reported more frequent insomnia-related thoughts than both control groups, and did not differ from each other. This suggests that poor sleep in the presence or absence of a co-morbid mental health problem is associated with more frequent pre-sleep thoughts about sleep and its consequences. Trauma-exposed and non-exposed controls did not differ in the frequency of either insomnia or trauma-related thoughts, suggesting that trauma-exposure alone is not related to increased pre-sleep thoughts of any kind.

Factor analysis showed that insomnia and trauma-related thoughts were reflected by separate factors. This suggests that in PTSD, although correlated, trauma-related pre-sleep thoughts are distinct from insomnia-related pre-sleep thoughts. However, this interpretation is limited by the small sample size.

In trauma-survivors the TTSI showed good sensitivity and specificity to detecting poor sleeper status. In trauma survivors both insomnia-related and trauma-related pre-sleep thoughts increased the likelihood of being a poor sleeper.

In sum, the pattern of group differences suggests similarities and differences between pre-sleep thought content in PTSD and insomnia, i.e. thoughts about sleep and its consequences are common to both groups, and those with PTSD reported additional thoughts about the trauma, as did those with depression (but to a lesser extent than those with PTSD). Both types of content are related to poor sleep in trauma survivors, and both are reported more frequently in PTSD than other clinical groups with poor sleep. These findings are consistent with evidence that similar mechanisms may underlie insomnia with or without a mental health problem (Kohn & Espie, 2005), and with suggestions that insomnia alone and in PTSD may have both similarities and differences (Ulmer, Edinger, & Calhoun, 2011). The TTSI may provide a brief, clinically useful tool to assess the type and frequency of trauma-related pre-sleep thoughts that might be keeping people with PTSD awake.

### 4.1. Limitations

The present study had a number of limitations. Firstly, measures of arousal and sleep were self-reported rather than objective. Further investigation is required to determine whether TTSI scores are also related to objective measures of poor sleep and physiological arousal. Secondly, insomnia and depression groupings were based on self-reports and the results may be different for individuals with a clinical diagnosis, particularly those seeking treatment, who may be more severe. Finally the analyses were limited by the smaller clinical group sample sizes. There is a need for further evaluation of the scale using multi-group confirmatory factor analyses to test factorial invariance across clinical groups and across clinical and non-clinical groups. Future research could investigate the psychometric properties further in clinical, ideally treatment-seeking, populations, with larger samples.

### 4.2. Future directions

A number of experts have recommended including measures of pre-sleep thoughts, behaviours and arousal with sleep outcome measures when investigating the experience of insomnia (Kohn & Espie, 2005) and the effects of treatment on sleep disturbances (Harvey, 2002). Therefore future studies investigating the effect of insomnia or PTSD treatments on sleep problems in PTSD could aim to include measures of insomnia-related (e.g. GCTI; see Espie et al. (2014) for a 9-item version) and trauma-related (e.g. TTSI) pre-sleep thoughts. This would help determine whether insomnia and PTSD treatments, both of which improve sleep in PTSD (e.g. Belleville, Guay, & Marchand, 2011; Lommen et al., 2015; Talbot et al., 2014; Ulmer et al., 2011), also produce change in pre-sleep thoughts. A possibility is that trauma-focused therapy for PTSD may improve trauma- but not insomnia-related pre-sleep thoughts, which could theoretically contribute to residual sleep problems after otherwise effective PTSD treatment (Gutner, Casement, Gilbert, & Resick, 2013; Zayfert & DeViva, 2004).

### 4.3. Conclusion

In conclusion, a brief measure to assess trauma-related pre-sleep thoughts was developed and validated. The TTSI had good psychometric properties, and discriminated people with PTSD from insomnia and depression, and was sensitive to detecting poor sleep in trauma survivors. The utility of the TTSI for measuring pre-sleep thought frequency and content in PTSD (and possibly in other stressor or trauma-related disorders such as depression) is supported, although replication of the psychometric properties in a clinical sample is required. Future studies could examine the contribution of pre-sleep thoughts to sleep problems in PTSD, and persistence after treatment.
